# Computational identification of potential modulators of heme-regulated inhibitor (HRI) for pharmacological intervention against sickle cell disease

**DOI:** 10.21203/rs.3.rs-3755458/v1

**Published:** 2023-12-15

**Authors:** Afolabi J. Owoloye, Samuel O. Olubode, Adewale Ogunleye, Emmanuel T. Idowu, Kolapo M. Oyebola

**Affiliations:** Mountain Top University; Adekunle Ajasin University Akungba; University of Würzburg; University of Lagos; Mountain Top University

**Keywords:** Sickle cell, Fetal hemoglobin, Heme-regulated inhibitor, Molecular docking, MMGBSA

## Abstract

**Background:**

Sickle cell disease (SCD) poses a significant health challenge and therapeutic approaches often target fetal hemoglobin (HbF) to ameliorate symptoms. Hydroxyurea, a current therapeutic option for SCD, has shown efficacy in increasing HbF levels. However, concerns about myelosuppression and thrombocytopenia necessitate the exploration of alternative compounds. Heme-regulated inhibitor (HRI) presents a promising target for pharmacological intervention in SCD due to its association with HbF modulation. This study systematically screened compounds for their potential inhibitory functions against HRI.

**Methods:**

Small-molecule compounds from 17 plants commonly utilized in traditional SCD management were subjected to *in silico* screening against HRI. Molecular docking was performed, and free binding energy calculations were determined using molecular mechanics with generalized born and surface area (MMGBSA). The lead compounds were subjected to molecular dynamics simulation at 100 ns. Computational quantum mechanical modelling of the lead compounds was subsequently performed. We further examined the pharmacodynamics, pharmacokinetic and physiological properties of the identified compounds.

**Results:**

Five potential HRI inhibitors, including kaempferol-3-(2G-glucosyrutinoside), epigallocatechin gallate, tiliroside, myricetin-3-O-glucoside, and cannabiscitrin, with respective docking scores of −16.0, −12.17, −11.37, −11.56 and 11.07 kcal/mol, were identified. The MMGBSA analysis of the complexes yielded free-binding energies of −69.76, −71.17, −60.44, 53.55, and − 55 kcal/mol, respectively. The identified leads were stable within HRI binding pocket for the duration of 100 ns simulation.

**Conclusions:**

The study successfully identified five phytoligands with potential inhibitory effects on HRI, opening avenues for their use as modulators of HbF in SCD patients. This finding holds promise for advancing treatment strategies in SCD. However, additional preclinical analyses are warranted to validate the chemotherapeutic properties of the lead compounds.

## Background

Sickle cell disease (SCD) is an inherited genetic disorder characterized by the abnormal shape of red blood cells (RBCs), leading to various complications and health problems [1, 2]. The condition arises from a mutation in one of the hemoglobin (Hb) genes, causing the substitution of the sixth amino acid of the β chains of the hemoglobin tetramer. This results in the replacement of the usual adult HbA with Hemoglobin S (HbS), where an uncharged valine amino acid replaces the negatively charged glutamic acid residue [3]. The absence of a negative charge at this site, coupled with the alteration in the Hb protein’s shape during deoxygenation, facilitates the cohesion of neighboring deoxyHbS molecules into polymers [2].

The β’Valine at position 6 of one β chain forms hydrophobic contacts with β’Phenyalanin at position 85 and β’Leucine at position 88 of the other β chain, leading to the formation of rigid, helical chains consisting of 14 members that are highly ordered [4]. These polymers have the potential to alter the morphology of red cells, resulting in the formation of sickles and other abnormal shapes. The mutation increases the propensity of HbS molecules to form polymers, particularly in conditions of dehydration or acidosis [5].

Addressing sickle cell disease (SCD) poses an urgent demand for innovative therapeutic strategies [6]. Hydroxyurea (HU), identified as a myelosuppressive agent, exhibits characteristics making it an ideal drug for SCD, delivering therapeutic benefits through diverse mechanisms of action [7]. Functioning as a potent ribonucleotide reductase (RR) inhibitor, HU reduces intracellular deoxynucleotide triphosphate pools, particularly impacting S-phase and inhibiting DNA synthesis, ultimately inducing cellular cytotoxicity [7]. The RR M2 subunit is a direct target of hydroxyurea [8]. Presumably, HU introduces sporadic cytotoxic suppression of erythroid progenitors and cell stress signaling with a daily dosage in SCD, influencing erythropoiesis kinetics and prompting the recruitment of erythroid progenitors characterized by elevated fetal hemoglobin (HbF) levels [8, 9]. However, an ongoing debate surrounds whether HU induces myelosuppression and/or thrombocytopenia [10]. While stem cell transplantation and gene therapies represent advanced solutions to SCD, they come with substantial risks, including potential host rejection and the risk of insertional mutagenesis [11]. Moreover, the associated costs and technical expertise required present further challenges [12]. Effectively managing SCD in resource-limited settings necessitates the development of novel, inexpensive antisickling drugs.

The severity of hemoglobinopathies like sickle cell disease (SCD) can be alleviated by increased fetal hemoglobin (HbF) levels, either due to genetic variations or therapeutic interventions with HbF inducers such as hydroxyurea (HU) [11, 13]. Consequently, a crucial therapeutic goal is to reverse the developmental transition from adult hemoglobin (HbA) to HbF. Recent studies have identified heme-regulated inhibitor (HRI), also known as eukaryotic translation initiation factor 2-alpha kinase 1 (EIF2αK1), as a novel regulator of HbF. HRI suppresses HbF by elevating ATF4 levels [14]. In vitro studies have demonstrated that the loss of HRI is well-tolerated in human erythroid cells [14, 15], suggesting it as a potential drug target for increasing HbF levels. Therefore, HRI stands out as a molecular target in the pathophysiology of SCD.

This study examined small-molecule compounds from plants traditionally used as anti-sickling treatments. The investigation utilized molecular docking, free binding energy calculation, molecular dynamics simulation, and pharmacokinetic analysis to predict the binding affinity, binding energy, stability, and toxicity levels of these small drug molecules.

## Methods

### Ligand harvest and preparation

A dataset of 2,274 chemical compounds from plants associated with folkloric management of SCD was sourced from the PubChem Database [16] in Structure Data File (SDF) format. To enhance the dataset’s structural representation and enable three-dimensional (3D) visualization, a meticulous preparation process was undertaken. Leveraging the advanced capabilities of the Ligprep tool [17] within the Schrödinger Suite, each compound was transformed to generate accurate and realistic 3D molecular structures using OPLS4 forcefield [3].

### Protein preparation

The structural model of the HRI protein was retrieved from the Protein Data Bank (PDB) database with the identifier AF-Q9BQI3-F1 [18]. Subsequently, a comprehensive preparation process was conducted using the Protein Preparation Wizard tool within the Schrödinger Suite. This meticulous step aimed to address potential anomalies and errors that may have been present in the computationally predicted structure. Common issues addressed included incomplete loops, missing hydrogen atoms, and overlapping atomic positions. By resolving these issues, the protein preparation process ensured the integrity and accuracy of the structural representation, mitigating any errors that could have arisen during the crystallization process.

### Grid generation

The SiteMap tool of the Schrödinger Suite was employed to assess the protein binding interface. A cubic grid box (30.1 Å) around the active site was generated to delineate and characterize the ligand binding cavity, enhancing the precision and depth of exploration.

### Molecular docking

The precise docking of prepared ligands into the binding site of the refined protein structure was carried out using the Glide tool [19] within the Schrödinger Suite. Both the standard precision (SP) and extra precision (XP) algorithms within the Glide tool were employed for a systematic assessment and analysis of ligand-protein interactions. The SP algorithm facilitated an initial high-throughput screening, while the XP algorithm, known for its enhanced accuracy, further refined the top-ranking poses generated by SP. This refinement process provided a more detailed and reliable depiction of ligand binding orientations [20]. By leveraging the capabilities of these algorithms, the docking process not only identified potential binding modes but also prioritized those with the highest likelihood of biological relevance.

### Prime MMGBSA

To assess the binding free energy of the selected complexes, the Prime MM-GBSA methodology [21] seamlessly integrated within the Prime module of the Schrödinger suite was employed. The computation of target-ligand complexes involved the implementation of the variable solvent generalized born (VSGB) solvation system and the OPLS4 forcefield, ensuring a robust and accurate representation of molecular interactions. The computational protocol further encompassed the incorporation of the rotamer search technique, coupled with the optimization achieved through the minimization of the sampling model [22]. These refinements contribute to a more exhaustive exploration of the conformational space and enhance the reliability of the binding free energy calculations [23]. The determination of the relative binding free energy was executed using the following equation:

ΔGbind=Gcomplex−(Gprotein+Gligand)

### ADMET

After conducting binding affinity and binding energy calculations, the identified lead compounds underwent a subsequent phase of absorption, distribution, metabolism, excretion and toxicity (ADMET) screening. To comprehensively assess their pharmaceutical viability, sophisticated tools such as the SwissADME and Pro-tox II servers were employed [23, 24]. These tools were utilized to extract crucial insights into the physicochemical properties, pharmacokinetic profiles, drug-likeness, and potential toxicity of the identified hits. The ADMET screening process plays a pivotal role in the drug discovery pipeline, allowing for a holistic evaluation of a compound’s absorption, distribution, metabolism, excretion, and toxicity [23]. This step is crucial for understanding the overall pharmaceutical viability and safety profile of the compounds in the context of drug development.

### Induced Fit Docking (IFD)

The induced fit docking (IFD) protocol was implemented using Maestro Schrödinger 12.9, focusing on the top five hit compounds. IFD, a dynamic docking methodology, introduces flexibility for both the ligand and its target receptor, thereby enhancing the accuracy of molecular docking predictions. This involves iterative docking of flexible ligands into a rigid receptor, followed by refinement of the protein’s active site to adopt the most favorable conformation for the ligand. The IFD panel within Schrödinger Maestro was employed following the methodology outlined by Kikiowo *et al*. [25].

For the selected (centroid) amino acid residues side chain, the standard IFD protocol was implemented with the OPLS4 force field in an implicit solvent model. Restrictions related to metal ions and hydrogen bonds were applied to both the initial stage and the re-docking procedures. Non-planar conformation on amide bonds and ring conformational sampling were penalized with an energy barrier of 2.5 kcal/mol incorporated into the IFD process. The receptor van der Waals scaling was set at 0.5, and the maximum poses for each ligand were limited to 20. Residues within 5 Å of the docked ligand were refined using the Prime Refinement approach. Receptor configurations that deviated below 30 kcal/mol from the set minimal energy structure were resubmitted for a final process of binding affinity calculation and scoring. In the subsequent docking stage, each compound was re-docked into all the modified low-energy receptor structures using the default Glide XP settings.

### MD simulation

To simulate the dynamic behavior of the biological environment, including lipid membranes and water molecules, molecular dynamics (MD) simulation was employed. Utilizing Newton’s equations, these simulations elucidate the motion and interactions of various entities, encompassing water, ions, small molecules, macromolecules, and more intricate systems [26]. In this study, a 100-nanosecond MD simulation was conducted using the Desmond software provided by Schrödinger [27]. The protein-ligand complexes selected for the MD simulations were based on earlier docking studies, providing a predictive perspective. The protein-ligand systems were prepared using the System Builder tool in Schrödinger suites (version 2021–2) [27]. The simulation utilized the OPLS4 force field, with the solvent model set as TIP3P in an orthorhombic box. Counter ions were added as needed to achieve a neutral model. NaCl (0.15 M) was introduced to mimic physiological conditions. The NpT ensemble was chosen for a comprehensive simulation, utilizing the Martyna–Tuckerman–Klein Barostat at a temperature of 300 K and atmospheric pressure.

Prior to the simulation, the model underwent relaxation, and the trajectory of each run was saved for analysis at intervals of 100 ns. The stability of the simulations was assessed by computing the root mean square deviation (RMSD) of the protein and ligand over time. Additionally, the root mean square fluctuation (RMSF) and protein-ligand interactions were studied to gain insights into the system’s behavior. This approach allowed for a detailed examination of the molecular dynamics and interactions within the simulated biological environment.

### Quantum chemical calculations

Quantum chemical calculations were carried out using Jaguar [28], a specialized quantum chemistry software seamlessly integrated into the Maestro platform. The molecular geometry underwent systematic optimization through the application of the B3LYP functional in conjunction with the 6–31G* basis set. This rigorous computational approach aimed to investigate essential molecular properties, such as the lowest unoccupied molecular orbital (LUMO), highest occupied molecular orbital (HOMO), bandgap energy (Eg), ionization potential (I), electron affinity (A), electronegativity (χ), hardness (η), electrophilicity (δ), and dipole moment (ω) of the compound [29]. The provided equations facilitated the determination of critical molecular parameters, providing insights into the electronic characteristics and overall behavior of the compounds [29].

The equations used for derivation are as follows:

Eg=ELUMO−EHOMO

I=−EHOMO

A=−ELUMO

χ=(I+A)/2

η=(I−A)/2

δ=1/η

These equations allowed the extraction of crucial information about the electronic properties of the compounds, contributing to a comprehensive understanding of their behavior.

## Results

### Molecular docking

**Figure 2:**
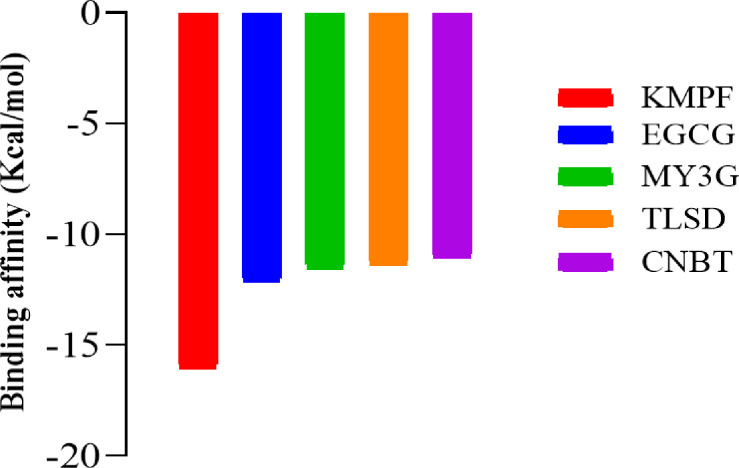
Binding affinity of the lead compounds with EIF2αK1 (AF-Q9BQI3-F1). KMPF = kaempferol-3-(2G-glucosylrutinoside) EGCG = epigallocatechin gallate, MY3G = myricetin-3-O-glucoside, TLSD = tiliroside, CNBT = cannabiscitrin

The inhibition of HRI was predominantly determined by the interaction of the ligands with the amino acid residues at the active site of the target protein. Figure 2 illustrates the interactions that significantly contribute to the inhibition. A comprehensive screening of 2,274 compounds from 12 plants used in the folkloric management of sickle cell disease against the target protein, HRI, led to the identification of five hits. These hits demonstrated excellent binding affinities, surpassing the binding affinity of hydroxyurea. The identification and validation process involved rigorous assessments using various parameters, ensuring the reliability of the selected compounds for further consideration in drug development.

### Kaempferol-3-(2G-glucosylrutinoside) with HRI

**Figure 3:**
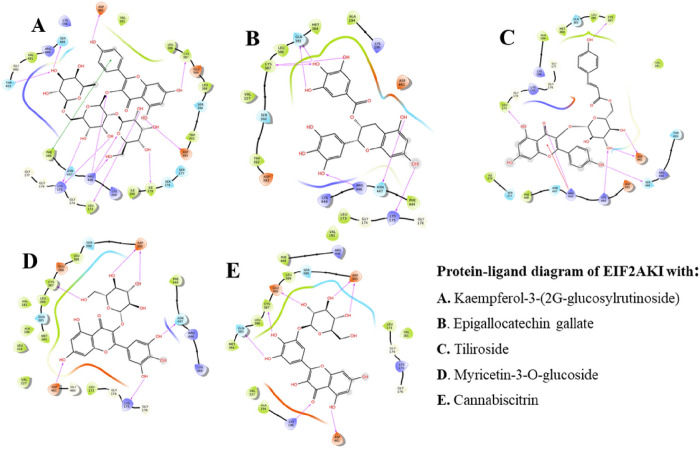
The 2D structures of interaction profile of EIF2αK1–ligand complexes after molecular docking studies. KMPF = kaempferol-3-(2G-glucosylrutinoside) EGCG = epigallocatechin gallate, MY3G = myricetin-3-O-glucoside, TLSD = tiliroside, CNBT = cannabiscitrin

Kaempferol-3-(2G-glucosylrutinoside) (KMPF), one of the identified hits, exhibited a notable binding affinity of −16.06 Kcal/mol. The 2D interaction analysis of the KMPF interaction with HRI, as illustrated in Fig. 3A, reveals a complex formed through several intermolecular interactions. The active domain’s amino acid residues engaged in hydrogen bonding with the ligand include Ser487, Asp461, Cys387, Asp393, Ile279, Leu173, Arg446 (triple bonds), Lys175 (double bonds), Phe449, and Thr493. In addition, a pi-cation bond with Phe449 was observed. The high binding affinity of KMPF can be attributed to the multitude of amino acids participating in the interaction with the ligand. The specific type of bonds formed, including hydrogen bonds and pi-cation bonds, further contributes to the strength of the interaction. Notably, the proximity of the bonds, with distances less than 3.0 Å, also enhances the robustness of the complex formed between KMPF and HRI.

### Epigallocatechin gallate with HRI

Epigallocatechin gallate (EGCG) demonstrated a robust binding affinity of −12.16 Kcal/mol in its complex formation with HRI. As illustrated in Fig. 3B, the amino acid residues in the active domain of HRI played a crucial role in forming a stable complex with EGCG. This interaction was predominantly characterized by hydrogen bonds, showcasing significant strength. Notably, the amino acid residues involved in this interaction were found exclusively within the active domain, indicating a focused and specific binding. The amino acid residues Gln385 and Cys387 were particularly noteworthy, forming double hydrogen bonds with distances less than 2.5 Å. Moreover, single hydrogen bonds were observed with Lys175, Arg446, and Asn447 residues, further contributing to the stability of the complex.

### Tiliroside with HRI

Tiliroside (TLSD), identified as a glycosyloxyflavone, exhibited a stable complex formation within the active site of the target protein, as illustrated in Fig. 3C. TLSD demonstrated a commendable binding affinity of −11.38 Kcal/mol in its interaction with the target protein. The intermolecular interactions between the amino acids of the target and TLSD were predominantly characterized by hydrogen bonds, with the addition of one pi-cation bond. All interactions within the 3Å range were considered strong. Asp461 formed a double bond with two hydroxyl groups of the compound, showcasing a robust interaction. Furthermore, single hydrogen bonds were observed with Cys387, Ser489, Lys444, and Leu173, while Arg446 formed one hydrogen bond and one pi-cation bond, further contributing to the stability of the complex.

### Myricetin-3-O-glucoside with HRI

The 2D representation of the Myricetin-3-O-glucoside-EIF2αK1 complex is shown in Fig. 3D. The Myricetin-3-O-glucoside (MY3G) was seen to be embedded deeply inside the active domain of the target with a binding affinity of −11.56 Kcal/mol. All the molecular interactions were found to be hydrogen bonds with less than 2.5 Å.

### Cannabiscitrin with HRI

Cannabiscitrin (CNBT), identified as a natural product present in Cannabis sativa and Ribes rubrum, exhibited a binding affinity of −11.07 kcal/mol with the target protein. The protein-ligand complex demonstrated a robust interaction, with the ligand snugly fitting into the binding pocket of the target protein, as depicted in Fig. 3E. The amino acid residue Asp393 played a key role in the interaction, forming a double hydrogen bond with the ligand at less than 2.5 Å. Besides, other amino acid residues, including Gln385, Cys387, Glu388, Asp 461, and Lys196, contributed to the stability of the complex by forming single hydrogen bonds. These interactions collectively underscore the strong binding and favorable fit of Cannabiscitrin within the target’s binding site.

### Binding free energy

**Figure 4:**
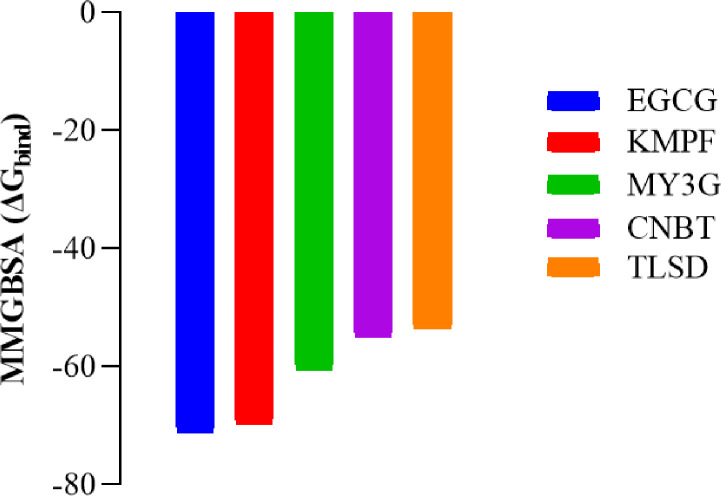
Prime/MMGBSA binding free energy (ΔGbind) of the lead compounds. KMPF = kaempferol-3-(2G-glucosylrutinoside) EGCG = epigallocatechin gallate, MY3G = myricetin-3-O-glucoside, TLSD = tiliroside, CNBT = cannabiscitrin

The molecular mechanics with generalized born and surface area solvation (MMGBSA) technique was employed to estimate the binding free energy of the protein-ligand complexes formed because of the ligands binding to the target protein. This calculation serves as a measure of the spontaneity of interactions between the ligand and the protein. The Prime MMGBSA tool within the Maestro Schrödinger suite was utilized for this purpose, known for its efficiency and reliability in calculating binding free energy [30]. The MMGBSA results for the complexes, as illustrated in Fig. 4, indicate that all the identified hit compounds exhibited stable binding affinities with comparatively high free binding energies. Among them, EGCG demonstrated the highest binding free energy at −71.17 kcal/mol. KMPF, despite having the highest docking score, showed a relatively lower binding free energy at −69.76 kcal/mol when compared to EGCG. The order of binding free energies for the other compounds is MY3G (−60.44 kcal/mol) > CNBT (−55.01 kcal/mol) > TLSD (−53.55 kcal/mol). These results provide insights into the energetics of the ligand-protein interactions, with EGCG standing out as having the most favorable binding free energy among the tested compounds.

### Induced fit docking

The docking scores of the complexes were validated using an IFD protocol, leveraging the Prime Glide and Refinement modules. The IFD protocol accurately predicts modes of ligand interaction and concurrent structural changes in the target receptor. Unlike standard precision and extra precision (SP and XP) virtual molecular docking studies, IFD allows the receptor motifs to modify their binding site to adjust to the conformation and binding algorithm of the ligand. This approach aims to address false positive results in SP and XP virtual screening by considering flexible receptor conformations.

The IFD results for the complexes, as summarized in Table 1, exhibited minimal deviation from the mean (mean ± standard error of the mean). KMPF obtained a score of −1237.49 ± 0.8, while EGCG showed − 1236.77 ± 0.39. TLSD, CNBT, and MY3G had scores of −1237.17 ± 0.42, −1231.88 ± 0.42, and − 1233.30 ± 0.46 kcal/mol, respectively. These results further validate the robustness of the docking scores obtained for the ligand-protein complexes, confirming the reliability of the virtual screening methodology employed.

### ADMET analysis

**Figure 5:**
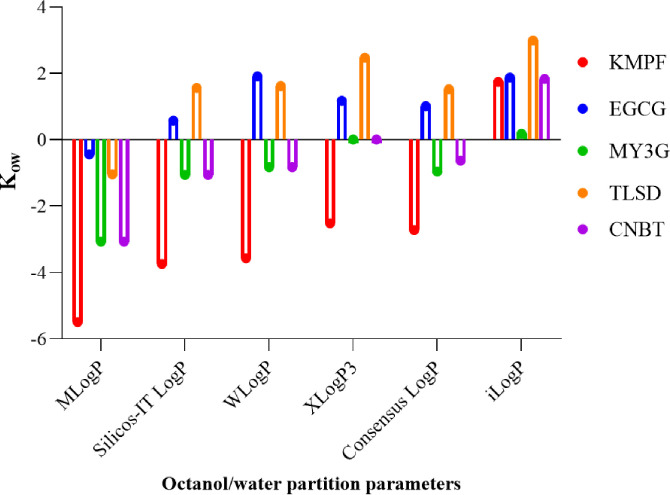
Octanol/water partition coefficient of the lead compounds (threshold: −6 to 4). KMPF = kaempferol-3-(2G-glucosylrutinoside) EGCG = epigallocatechin gallate, MY3G = myricetin-3-O-glucoside, TLSD = tiliroside, CNBT = cannabiscitrin

To analyze the pharmacokinetics of the lead compounds, the SwissADME webserver was utilized, employing existing models to predict their pharmacokinetic properties. The analysis revealed that none of the five lead compounds could cross the blood-brain barrier, indicating that these compounds are not capable of interfering with the metabolic activities of the central nervous system. Pharmacokinetic activities involve the interaction of compounds with cytochrome P450 (CYP450) substrates. The CYP450 superfamily, a group of isoenzymes, plays a crucial role in drug elimination through metabolic biotransformation [31]. Inhibition of these isoenzymes is a key factor in drug-drug interactions linked to pharmacokinetics. Surprisingly, none of the compounds were found to be substrates for any of the CYP450 isoenzymes (CYP1A2, CYP2C19, CYP2C9, CYP2D6, and CYP3A4). In addition, none of the compounds were identified as P-glycoprotein substrates. This information contributes valuable insights into the pharmacokinetic profile of the lead compounds, supporting their potential as drug candidates. The lipophilicity of a potential drug is a crucial factor as it influences the drug’s ability to pass through cell membranes, impacting its biological activity [32]. The octanol/water partition coefficient is a key physicochemical parameter in drug discovery, design, and development. In this study, the investigated compounds were found to be solubilized in a two-solvent system at a pH ensuring the neutral form of the compound (Fig. 5). This enabled the measurement of equilibrium concentrations in water and n-octanol, respectively.

Using the SwissADME server, various lipophilicity parameters were predicted, including ilogP, XlogP3, WlogP, MlogP, SILICOS-IT logP, and the consensus logP. None of the compounds crossed the benchmark threshold from − 6 to 4. Although KMPF showed a low MlogP (−5.49), all other lipophilic parameters were moderate. For oral bioavailability and absorption, good aqueous solubility is essential. The analysis of aqueous solubility (log S) of the lead compounds revealed that four compounds exhibited very good aqueous solubility properties (−3.5 < log S < −2.5), while TLSD demonstrated good solubility (log S = −4.90). These findings contribute to the understanding of the compounds’ physicochemical properties, which are crucial for their potential as drug candidates.

### MD simulation

The MD simulation was conducted for the protein-ligand complexes using the Desmond tool of the Maestro Schrodinger suites (version 2021) [27]. The chosen complexes exhibited high binding affinity, stable IFD conformations, and favorable pharmacokinetic and pharmacodynamic properties. The analysis aimed to evaluate the system’s stability under solvent and physiological conditions, including an apo-state (unbound) of the protein for comparison. The RMSD and RMSF of the complexes and the apo-state were estimated. The RMSD plot of the protein Cα showed variations among the complexes. The Cα of CNBT exhibited fluctuations throughout the simulation period, reaching stability after an initial fluctuation. TLSD, EGCG, and MY3G complexes attained stability within the first seconds, maintaining relatively constant RMSD values. The kaempferol-3-(2G-glucosyrutinoside) (KMPF) complex showed fluctuations up to 20 ns, followed by a gradual stabilization.

The ligand RMSD of the complexes was generally lower than the protein Cα RMSD. MY3G and kaempferol-3-(2G-glucosyrutinoside) exhibited the least ligand RMSD, with relatively stable values. TLSD, CNBT, and EGCG complexes reached stable ligand RMSD after initial fluctuations. The change in RMSF was calculated to estimate internal fluctuations in the protein and ligands. MY3G showed the least fluctuation, with notable fluctuations in residue positions 169 to 172. TLSD exhibited minimal fluctuation except for residues 175 to 182. CNBT, EGCG, and KMPF complexes showed significant fluctuations in specific residue positions. The protein SSE analysis indicated a stable composition of alpha-helices and beta-strands throughout the simulation period for the 5 complexes. The SSE composition revealed a higher proportion of alpha-helices (38.5%) compared to beta-strands (8.5%). Overall, the MD simulation provided insights into the dynamic behavior and stability of the protein-ligand complexes under physiological conditions, contributing valuable information for drug development studies.

### Protein-Ligand Contact

**Figure 8:**
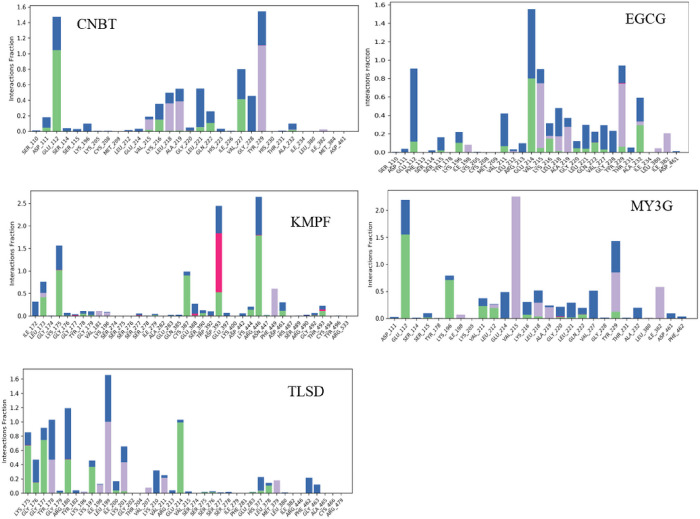
Protein-ligands contacts monitored throughout 100 ns simulation run time of EIF2ΑK1 complex with the lead compounds. KMPF = kaempferol-3-(2G-glucosylrutinoside) EGCG = epigallocatechin gallate, MY3G = myricetin-3-O-glucoside, TLSD = tiliroside, CNBT = cannabiscitrin

The target-ligand interactions were monitored during the simulation. The amino acid residues observed in the binding of the protein show predominantly noncovalent hydrogen with the ligands. The amino acid residues that interacted with the ligand through hydrogen bonds were Asp111, Ser115, Ile173, Lys175, Lys196, Val211, Glu214, Val215, Lys216, Leu218, gly220, Leu221, Gln222, Val227, Tyr229, Ala232 (Fig. 8; Additional File 1). Water bridge interactions were also observed in the five complexes. Hydrophobic interactions with similar amino acid residues were also observed to play a major in four of the five complexes (EGCG, TLSD, MY3G, and CNBT). Meanwhile, ionic bonds were observed in kaempferol-3-(2G-glucosyrutinoside) with six amino acid representatives. The ionic bonds that were observed in the remaining four complexes are negligible.

## Discussion

While the fundamental causes and molecular irregularities of sickle cell disease are well-documented, therapeutic interventions have been limited. However, there have been advancements in the treatment of SCD with HU [33]. Despite these successes, a notable portion of SCD patients experience insufficient responses, prompting concerns about the long-term safety of this cytotoxic drug [34]. High-throughput screening efforts have explored alternatives to hydroxyurea for enhancing fetal hemoglobin (HbF) production which plays a role in preventing hemoglobin S (HbS) polymerization [35]. A recent study evaluated over 170,000 compounds using modified K562 reporter cells. However, the potent stimulatory effects were associated with significant cytotoxicity [36, 37]. The objective of this current study was to identify novel anti-sickling drug candidates that promote fetal hemoglobin (HbF) production with minimal or no adverse pharmacokinetic effects, suitable for combination therapy with hydroxyurea. This *in silico* study encompasses experimental procedures evaluating molecular interactions, binding affinity, pharmacokinetic and pharmacodynamic profiles, molecular dynamic simulation, and the inhibitory potential of novel HRI modulators. The study involved screening a library of 2,274 compounds against HRI, resulting in the identification of five potential inhibitors.

The top five compounds exhibited favorable pharmacokinetic and pharmacodynamic properties, binding free energy, and induced fit docking scores, interacting with the target in the binding pocket through common amino acid residues (Arg446, Asn447, Asp393, Asp461, Cys387, Gln385, Leu173, and Leu175). KMPF demonstrated the highest binding affinity (−16.06 kcal/mol), followed by EGCG (−12.165 kcal/mol), TLSD (−11.38 kcal/mol), MY3G (−11.56 kcal/mol), and CNBT (−11.07 kcal/mol). The robust binding of KMPF may be attributed to strong hydrogen bonds with specific amino acid residues [38]. These lead phyto-ligands displayed robust intermolecular interactions with the target, making them effective inhibitors. Combinatorial therapy with HbF pharmacologic inducers and HRI inhibition, as well as the potential synergy with hydroxyurea, was discussed. Molecular dynamics simulations and binding free energy calculations using MM-GBSA indicated stability in the ligand-protein complexes, with EGCG forming the most stable complex.

The study recognized the limitations associated with the rigid receptor assumptions in Glide docking, underscoring the significance of IFD to accommodate conformational changes. Notably, the IFD scores for the five complexes demonstrated a level of stability. Molecular dynamics simulations further revealed overall stability in both protein Cα and ligand RMSD, with some initial fluctuations. The RMSD trajectories of the complexes indicated stability after the initial frames, suggesting a confined environment for the small molecules within the binding domain of HRI. Despite an initial significant shift, CNBT eventually stabilized. The RMSF analysis offered valuable insights into the stability of the complex binding site, highlighting that MY3G, TLSD, and KMPF maintained consistent interactions.

Intermolecular interactions during MD simulations, including hydrogen bonds and hydrophobic interactions, were observed for all compounds. Electronic properties such as EHOMO, ELUMO, band gap, ionization potential, and electron affinity were analyzed, providing valuable information for drug development. The compounds C1 to C5 exhibited smaller band gaps, suggesting higher reactivity, with C5 identified as the most reactive. However, higher reactivity could pose challenges such as non-specific binding or off-target effects. The computed electronegativity (χ) values provide insights into the compounds’ abilities to attract electrons within each molecule. C3 stood out with the highest electronegativity at 0.136, indicating a stronger electron-attracting capacity. C1, C2, and C4 exhibited similar electronegativity values around 0.12, suggesting a moderate electron-attracting capability. C5 closely followed, while C6 had the lowest electronegativity at 0.084, indicating a lesser tendency to attract electrons. These values offer valuable information about the influence of electron density distribution on chemical behavior and interactions. Hardness (η) values are crucial for understanding a compound’s resistance to deformation or changes in electron density upon electron addition or removal [39]. C6 showed the highest hardness at 0.219, indicating a relatively rigid molecular structure with low electron deformability. Conversely, C5 had the lowest hardness at 0.069, suggesting a more flexible molecular structure. The hardness values of C1 to C4 fell within this range, indicating varying degrees of resistance to electron perturbations [39].

Electrophilicity (δ) values reflect the compounds’ tendency to accept electrons and act as electrophiles in chemical reactions [40]. C6 exhibited the lowest electrophilicity at 4.566, indicating a relatively lower affinity to accept electrons. In contrast, C5 had the highest electrophilicity at 14.492, suggesting a stronger tendency to accept electrons. This information is crucial for predicting reactivity in chemical reactions, especially in drug development [40]. The computed dipole moment (ω) quantifies the separation of positive and negative charges in a molecule, providing insight into its overall polarity [41]. C3 displayed the highest dipole moment at 0.256, indicating substantial charge separation and heightened molecular polarity. C1, C2, and C4 presented moderate dipole moments (0.173 to 0.194), signifying a balanced charge distribution [42]. C5 closely followed this trend, while C6 featured the lowest dipole moment at 0.032, indicating a more symmetrical charge distribution. All lead compounds appeared more reactive compared to the standard compound, with C5 identified as the most reactive. However, it is crucial to emphasize the need for further optimization to refine its properties and ensure its suitability as a pharmaceutical agent. *In vitro* and *in vivo* validation will provide essential data regarding the compound’s behavior in biological systems, its efficacy, and potential side effects. This comprehensive evaluation is essential before considering the lead compound for further development and potential use in medical applications.

## Conclusions

The utilization of *in silico* screening, molecular docking, free binding energy calculations (MMGBSA), molecular dynamics simulations, and computational quantum mechanical modeling has led to the identification of five potential HRI inhibitors. These compounds exhibit notable docking scores and favorable free-binding energies. The stability of the identified lead compounds within the HRI binding pocket adds credibility to their potential as therapeutic candidates. This research provides a solid foundation for future experimental studies and clinical investigations aimed at validating the efficacy and safety of the identified compounds as potential alternatives or supplements to existing therapies for sickle cell disease. The pursuit of novel therapeutic avenues, as illuminated by this study, holds promise for enhancing the treatment landscape and improving the quality of life for individuals living with SCD.

## Figures and Tables

**Figure 1:**
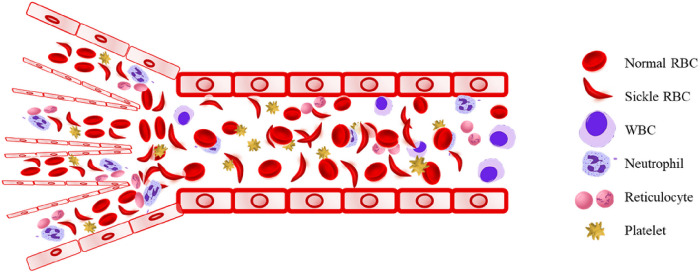
Blood vessel showing normal and sickled red blood cells together with other components of the blood.

**Figure 6:**
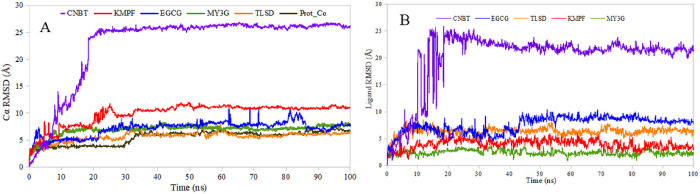
A graphical representation of the evolution of the RMSD conformational trend at the time of the MD simulation. (A) Cα apo state and the lead compounds. (B) Ligand RSMD. KMPF = kaempferol-3-(2G-glucosylrutinoside) EGCG = epigallocatechin gallate, MY3G = myricetin-3-O-glucoside, TLSD = tiliroside, CNBT = cannabiscitrin

**Figure 7:**
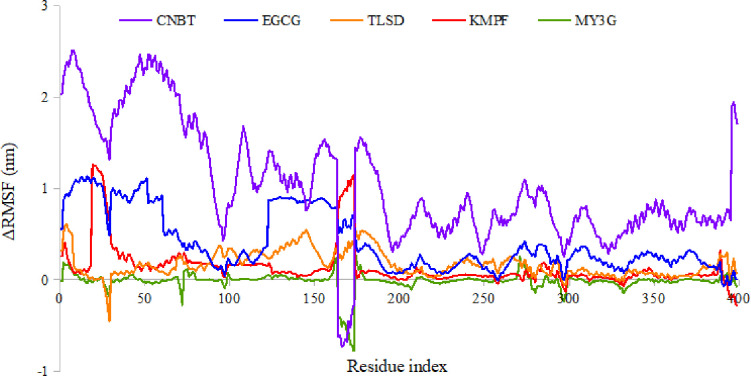
A graphical representation of the ΔRMSF of the lead compounds with respect to apo RMSF. KMPF = kaempferol-3-(2G-glucosylrutinoside) EGCG = epigallocatechin gallate, MY3G = myricetin-3-O-glucoside, TLSD = tiliroside, CNBT = cannabiscitrin

**Table 1:** The induced fit docking score and pharmacokinetic properties of the test compounds

Comp	Binding Affinity (kcal/mol)	IFD (kcal/mol)	BBB permeant	Pgp substrate	CYP1A2 inhibitor	CYP2C9 inhibitor	CYP2C19 inhibitor	CYP2D6 inhibitor	CYP3A4 inhibitor
KMPF	16.06	−1237.49±0.8	No	No	No	No	No	No	No
EGCG	−12.16	−1236.77±0.39	No	No	No	No	No	No	No
TLSD	−11.38	−1237.17±0.42	No	No	No	No	No	No	No
CBNT	−11.07	1231.88±0.42	No	No	No	No	No	No	No
MY3G	−11.56	1233.30±0.46	No	No	No	No	No	No	No

KMPF = kaempferol-3-(2G-glucosylrutinoside) EGCG = epigallocatechin gallate, MY3G = myricetin-3-O-glucoside, TLSD = tiliroside, CNBT = cannabiscitrin

## List Of Abbreviations

ADMET: Absorption, Distribution, Metabolism, Excretion, Toxicity
CNBT: Cannabiscitrin
EGCG: Epigallocatechin Gallate
Hb: Hemoglobin
HbF: Fetal Hemoglobin
HbS: Hemoglobin S
HOMO: Highest Occupied Molecular Orbital
HRI: Heme-Regulated Inhibitor
HU: Hydroxyurea
IFD: Induced Fit Docking
KMPF: Kaemferol-3-(2G-Glucosyrutinoside)
LUMO: Lowest Unoccupied Molecular Orbital
MD: Molecular Dynamics
MMGBSA: Molecular Mechanics with Generalized Born and Surface Area
MY3G: Myricetin-3-O-glucoside
PDB: Protein Data Bank
RBCs: Red Blood Cells
RMSD: Root Mean Square Deviation
RMSF: Root Mean Square Fluctuation
RR: Ribonucleotide Reductase
SCD: Sickle Cell Disease
SDF: Structured Data File
SP: Standard Precision
SSE: Secondary Structure Elements
TLSD: Tiliroside
VSGB: Variable Solvent Generalized Born
XP: Extra Precision
